# Prevalence of Psychological Trauma and Association with Current Health and Functioning in a Sample of HIV-infected and HIV-uninfected Tanzanian Adults

**DOI:** 10.1371/journal.pone.0036304

**Published:** 2012-05-14

**Authors:** Brian W. Pence, Kristen Shirey, Kathryn Whetten, Bernard Agala, Dafrosa Itemba, Julie Adams, Rachel Whetten, Jia Yao, John Shao

**Affiliations:** 1 Center for Health Policy & Inequalities Research, Duke Global Health Institute, Department of Community and Family Medicine, Duke University, Durham, North Carolina, United States of America; 2 Duke Global Health Institute, Departments of Psychiatry and Medicine, Duke University, Durham, North Carolina, United States of America; 3 Tanzania Women Research Foundation, Moshi, Tanzania; University of Cape Town, South Africa

## Abstract

**Background:**

In high income nations, traumatic life experiences such as childhood sexual abuse are much more common in people living with HIV/AIDS (PLWHA) than the general population, and trauma is associated with worse current health and functioning. Virtually no data exist on the prevalence or consequences of trauma for PLWHA in low income nations.

**Methodology/Principal Findings:**

We recruited four cohorts of Tanzanian patients in established medical care for HIV infection (n = 228), individuals newly testing positive for HIV (n = 267), individuals testing negative for HIV at the same sites (n = 182), and a random sample of community-dwelling adults (n = 249). We assessed lifetime prevalence of traumatic experiences, recent stressful life events, and current mental health and health-related physical functioning. Those with established HIV infection reported a greater number of childhood and lifetime traumatic experiences (2.1 and 3.0 respectively) than the community cohort (1.8 and 2.3). Those with established HIV infection reported greater post-traumatic stress disorder (PTSD) symptomatology and worse current health-related physical functioning. Each additional lifetime traumatic experience was associated with increased PTSD symptomatology and worse functioning.

**Conclusions/Significance:**

This study is the first to our knowledge in an HIV population from a low income nation to report the prevalence of a range of potentially traumatic life experiences compared to a matched community sample and to show that trauma history is associated with poorer health-related physical functioning. Our findings underscore the importance of considering psychosocial characteristics when planning to meet the health needs of PLWHA in low income countries.

## Introduction

In high income nations, studies of people living with HIV/AIDS (PLWHA) have consistently demonstrated a high lifetime prevalence of traumatic life experiences. [1] In a large US cohort of women with or at high risk for HIV, 66% had experienced domestic violence and 31% percent had experienced sexual abuse during childhood.^2^ These numbers are substantially higher than the general population lifetime prevalence of domestic violence (25%) and childhood sexual abuse (13%). [2], [3] In a large cohort of HIV patients from the US Deep South, 30% of participants reported sexual abuse and 20% reported physical abuse during childhood; overall, 91% of respondents reported at least one potentially traumatic experience during their lifetimes. [4], [5].

In recent years, increasing attention has turned to the adverse behavioral and health consequences of trauma histories in PLWHA. [6], [7] A history of potentially traumatic life events has been associated with development of post-traumatic stress disorder (PTSD), [8]–[13] depression, [14]–[18] and substance abuse, [12], [14], [19]–[21] as well as increased high risk sexual and drug use behaviors that increase the risk of becoming infected, and transmitting infection to others. [14], [22]–[28] Though rates of PTSD have not been studied in large cohorts, existing data, predominately from high income countries, suggest that PTSD is highly prevalent among PLWHA. In a study of 350 county-based HIV primary care clinic patients, 34% met screening criteria for PTSD. [29] A recent meta-analysis of 5,930 HIV positive women demonstrated rates of probable PTSD of 30%, which is five times higher than the rates of PTSD among women in the general U.S. population. [30] Moreover, those with a history of traumatic experiences have lower medication adherence, greater emergency department utilization, faster progression to AIDS, and higher mortality rates. [31]–[34].

A history of traumatic experiences also predicts poorer current health-related physical functioning in PLWHA. [31] Current physical functioning is an important outcome to consider in its own right due to the correlation of lower physical functioning with lower CD4 counts, higher viral loads, and reduced survival rates. [35]–[38] Given the chronic nature of HIV infection, physical functioning may have important implications for pain, ability to work, and activities of daily living over a period of many years.

Nearly all research on the impact of past trauma on current health and functioning for PLWHA has taken place in high income nations. In sub-Saharan Africa, home to two thirds of the world’s population of PLWHA, [39] there is limited data about exposure to potentially traumatic events or their physical and mental health sequelae. Among a cohort study of 44 HIV positive patients in a genitourinary medicine clinic in the Gambia, 43.2% endorsed PTSD symptoms. [40] A South African study conducted to validate the use of the 17 item Posttraumatic stress Diagnostic Scale (PDS) in a South African patient population surveyed a convenience sample of 85 recently diagnosed HIV positive patients. Among this sample, 44% met diagnostic criteria for current PTSD [41]and 54% met criteria for lifetime prevalence of PTSD. [42] Outside of this South African study, data are sparse regarding not only the prevalence of traumatic exposure in PLWHA, but also the relationship between traumatic exposure and current mental health and health-related physical functioning.

With the rapid expansion of access to antiretroviral therapy in low income nations, it is critically important to understand the prevalence of trauma history in PLWHA in the region as well as its influence on mental health and functioning. Accordingly, the purpose of the present paper is to describe the lifetime prevalence of potentially traumatic experiences in a sample of HIV-positive and HIV-negative Tanzanian adults and to examine associations between lifetime trauma history, current mental health, and health-related physical functioning.

## Methods

The Coping with HIV/AIDS in Tanzania (CHAT) Study is an observational cohort study designed to explore the longitudinal relationships between psychosocial characteristics, HIV medication adherence, and health outcomes among HIV-positive individuals in Tanzania. This study and all study activities were specifically approved by the Kilimanjaro Christian Medical Center Institutional Review Board in Tanzania and the Duke University Health System Institutional Review Board in the United States, and written informed consent was obtained from all participants.

From November 2008 to October 2009, the CHAT Study recruited 1,197 participants to form five distinct cohorts: patients with established HIV infection receiving care at the regional tertiary referral hospital (KCMC, clinic census ∼1400; sample n = 228); patients with established HIV infection receiving care at the local public hospital (clinic census ∼2700; sample n = 271); individuals newly diagnosed with HIV at voluntary counseling and testing (VCT) sites (n = 267); individuals testing HIV-negative at VCT sites (n = 182); and a random sample of adults from the surrounding community (n = 249).

Specific recruitment procedures were as follows. For the two clinical cohorts, any patient aged 18–65 residing in Moshi Urban and Hai districts of the Kilimanjaro region with plans to stay in the region for the foreseeable future was eligible to participate. Due to staffing and interview length, a maximum of 3 participants/clinic day could be enrolled at each clinic. At both clinics, it was estimated that at least 30% of patient visits were unscheduled, and there was a wide variation in the number of patients presenting on any given clinic day. Therefore, patients were selected by a random time point system. In order to construct the selection parameters for the random time selection, clinic flow was observed over the period of one week. From that week, the earliest arrival time of the second patient of the day and the latest arrival time of the next-to-last patient of the day were designated as the beginning and ending times for the time point sampling. Three minutes from the clinic day were randomly selected, with probability of selection proportional to the expected number of patients in a given time interval. The selected minutes for each day were distributed to the clinic nurses and programmed into alarms placed at the nursing triage station. After an alarm went off, the next patient in line for triage was screened for eligibility and, if eligible, read a standardized brief description of the research. Patients who were interested and desired further information underwent the full consent process with a research staff. If the selected patient declined or was ineligible, the next patient was approached as a replacement.

The cohorts of individuals newly testing HIV-positive and -negative at VCT sites were recruited as follows. All people 18–65 years old and presenting for testing at 4 VCT sites in Moshi Urban were eligible for enrollment. Every eligible client receiving a positive HIV test was invited to participate, and after every other person testing positive, the next client testing negative was also invited. If a client refused to participate the next client was asked to replace them. Clients were invited after receiving the results of their HIV test. They either completed the interview immediately (38% of those testing positive and 58% of those testing negative) or made an appointment to return for the interview (within 7 days: 49% of those testing positive and 36% of those testing negative; more than one week later: 13% of those testing positive and 6% of those testing negative). Although the large majority of clients at these VCT sites came on their own, occasionally couples presented for testing together; of 449 total participants enrolled from VCT sites, 3 couples were enrolled.

The community sample was recruited from households in the three districts corresponding to the majority of the catchment areas of the 4 VCT centers: Moshi Urban, Moshi Rural, and Hai districts. From the list of streets in each district, 25% of the streets were randomly selected (Moshi Urban –15 out of 60 streets, Moshi Rural –7 out of 30 streets and Hai –3 out of 10 streets). Street Leaders (each street has a designated Street Leader) were approached for lists of households on that street and the respective heads of those households. Ten households from each street were then randomly selected. The head of each selected household was then asked if the household would participate in the study. If the head of the household agreed, a census of household members between 18 and 65 years was taken. All those listed in the census register were asked if they would like to participate and of those who agreed, one was randomly selected through a randomization schedule. Recruitment for the community sample was performed without knowledge of HIV status.

Participants complete in-person interviews every six months with trained local interviewers not previously known to the participants; these interviews last approximately one hour. HIV-infected participants who have initiated clinical care also complete clinical exams and provide blood for CD4 counts and HIV RNA viral loads on the same schedule. All interviews are conducted in Swahili, with instruments translated from and back-translated to English to confirm the validity of the translation. The present manuscript focuses on the baseline in-person interview only. The analyses exclude the cohort from the local public hospital, for whom the questions about trauma history at baseline were administered inconsistently.

### Measures

Participants self-reported sociodemographic information including age, gender, marital status, highest level of completed education, religion, household assets, and tribe. *Overall health-related physical functioning* was assessed with the Short Form (SF)-8, a validated shortened version of the extensively used SF-36. [43], [44] The Physical Composite Score (PCS) was computed according to standard methodology; this score is a weighted average of the SF-8 items with heaviest weights given to questions focusing on overall perception of physical health, healthy physical functioning (e.g., degree to which health interferes with walking and lifting), functioning without bodily pain, and healthy role functioning (e.g., extent to which health limits work and activities). The score can range from 0–100, with higher scores indicating better health-related physical functioning and 10 units representing one standard deviation in the US normative population. [45].

Depressive severity was assessed with the Patient Health Questionnaire-9 (PHQ-9), a widely used depression case identification tool which has been validated in African populations[46], [47] and has a possible range of 0–27 with higher scores indicating greater depressive severity. [48], [49] *Post-traumatic stress disorder (PTSD) symptom severity* was assessed with the PTSD Symptoms Checklist (PCL) based on DSM-IV criteria that include re-experiencing a traumatic event, numbing/avoiding, and hyperarousal symptoms. This scale has strong reported reliability, and correlates highly with a clinician-administered PTSD measure. [50], [51].

Potentially traumatic experiences were defined, for the purposes of this study, as events that would satisfy criterion A(1) of the definition of PTSD in the Diagnostic and Statistical Manual of Mental Disorders (DSM-IV –TR). [52] The number of categories of lifetime potentially traumatic events was measured with a questionnaire, adapted from prior research by Leserman and colleagues, which included detailed questions about sexual abuse and severe physical trauma (age, number of experiences, perpetrator, etc.), a standardized assessment of childhood physical and emotional neglect, [53] and individual questions asking about other potentially traumatic experiences. [31], [53]–[56] Sexual abuse was defined in the analysis to include sexual experiences (e.g., touching, intercourse) where force or threat of force was used; however, in children (before the age of puberty) the threat of force or harm was implied by a 5-year age differential between the victim and perpetrator. Physical abuse was defined as incidents separate from sexual abuse that were perceived to be life threatening (being physically attacked with the intent to kill or seriously injure), and other physical abuse (being beaten, hit, kicked, bit, or burned). Childhood physical and emotional neglect was measured with the Childhood Trauma Questionnaire and scored using the cutoffs suggested by Bernstein and Fink for moderate physical neglect (≥9) and moderate emotional neglect (≥12). [53] Other potential traumas before age 18 were parental alcohol/drug abuse, depression, suicide or attempted suicide; imprisonment of a parent; domestic violence in the home; being placed in reform school, prison or jail, or foster or adoptive care; death of an immediate family member; and having a life-threatening illness or injury not related to HIV. Lifetime potential traumas included murder or death by trauma of a close family member, death of a child, and death of a spouse/partner. Participants were assigned a score from 0 to 15 reflecting the number of types of potentially traumatic events experienced in their lifetime. This specification of the number of types of potentially traumatic events experienced has been used widely and has been associated with multiple negative health outcomes. [31], [34], [57]–[59].

Recent stressful life events were measured with a modified version of the Life Events Survey (LES)[60], [61] to measure the occurrence of stressful events in the 6 months preceding the baseline interview. Only those events considered to be moderately to severely stressful based on previous studies with interviewer-based objectively rated stresses were included. [31], [62] Moderate stressors included experiences such as relationship difficulties; death or serious illness of a close friend or extended family member; employment difficulties (e.g. loss of job); and non-HIV-related serious illnesses, injuries, and accidents. Severe stressors included divorce/separation, death or illness of an immediate family member, major financial problems (e.g., loss of home), more than a week in prison, and sexual and physical assault.

### Statistical Analyses

Ordinary least squares regression was used to assess the association of measures of potentially traumatic and stressful life experiences with three measures of mental health and health-related physical functioning at the time of the baseline visit: PTSD symptom severity, depressive symptom severity, and SF-8 health-related physical functioning composite score. We included fixed effects for the four cohorts in each model. We examined whether the associations of life events with outcomes varied across the four cohorts by jointly testing a set of interaction terms between the site fixed effects and the life events measure in each model. We assessed the appropriateness of a linear specification of each continuous life events measure by testing the significance of adding a quadratic term.

We hypothesized that lifetime potentially traumatic events might influence current PTSD symptomatology through recent stressful life events and depressive symptoms, and that lifetime potentially traumatic events might influence current health-related physical functioning through recent stressful life events depressive symptoms, and PTSD symptoms. To explore these hypotheses for the outcome of PTSD symptomatology, we first built a model that included the continuous measure of lifetime potentially traumatic events as well as site fixed effects and sociodemographic variables as covariates (Model 1). Model 2 included all Model 1 variables as well as the measure of recent stressful life events, and Model 3 included all Model 2 variables as well as depressive symptoms. For the outcome of health-related physical functioning, Models 1–3 were specified similarly, and we added a Model 4 which included all Model 3 variables as well as PTSD symptomatology. We assessed whether these variables mediated the association of lifetime potentially traumatic events with current PTSD symptoms or health-related physical functioning by examining shifts in the magnitude of the coefficient for lifetime potentially traumatic events as the hypothesized mediators were progressively added to the model.

**Table 1:** Characteristics of Sample.

	Mean (SD) or n (%)			
Characteristic	Community	Newly tested HIV-	Newly diagnosed HIV+	Established HIV+
Sample size	249	182	267	228
Age (range: 18–69)	39.6 (12.3)	32.2 (10.2)**	37.5 (9.0)*	42.6 (8.1)**
*Gender*				
Male	100 (40.2)	92 (50.8)*	84 (31.6)*	76 (34.1)
Female	149 (59.8)	89 (49.2)*	182 (68.4)*	147 (65.9)
*Marital status*				
Married or cohabitating	176 (70.7)	81 (44.8)**	110 (41.4)**	87 (39.0)**
Never married	37 (14.9)	71 (39.2)**	46 (17.3)**	33 (14.8)**
Widowed	19 (7.6)	11 (6.1)**	43 (16.2)**	65 (29.1)**
Divorced	17 (6.8)	18 (9.9)**	67 (25.2)**	38 (17.0)**
*Highest level of education:*				
None	3 (1.2)	4 (2.2)	13 (4.9)**	9 (4.0)
Primary	191 (76.7)	133 (73.5)	218 (82.0)**	154 (69.1)
Secondary	45 (18.1)	40 (22.1)	34 (12.8)**	50 (22.4)
University	10 (4.0)	4 (2.2)	1 (0.4)**	10 (4.5)
Household asset score (range: 0–5)	2.3 (1.6)	2.1 (1.4)	2.0 (1.4)	2.2 (1.3)
*Physical and mental health*				
SF-8 health-related physical functioning (range: 0–100)	50.0 (8.6)	50.3 (9.4)	44.0 (11.5)**	45.2 (9.3)**
PHQ-9 depression score (range: 0–27)	4.0 (5.4)	3.9 (5.2)	7.6 (6.9)**	4.8 (5.5)
PTSD symptom severity (range: 17–85)	22.2 (7.5)	25.3 (7.2)**	27.9 (8.4)**	23.9 (7.1)*
Number of types of potentially traumatic experiences				
Childhood (range: 0–7)	1.8 (1.4)	1.5 (1.4)*	1.7 (1.4)	2.1 (1.5)*
Lifetime (range: 0–8)	2.3 (1.6)	1.9 (1.6)**	2.2 (1.6)	3.0 (1.9)**
Number of stressful life events, past 6 mo. (range: 0–14)	4.2 (2.8)	3.2 (2.5)**	2.9 (2.4)**	4.1 (2.4)

* p<0.05,

** p<0.01 comparing each cohort to the Community cohort using a Chi-square test (categorical variables) or t-test (continuous variables).

## Results

### Sample Description

The four cohorts included 926 participants (249 community members, 182 newly tested HIV-negative, 267 newly tested HIV-positive, and 228 with established HIV infection) (Table 1). Response rates (the proportion of all those eligible and approached who provided informed consent) were 98% (community), 88% (newly tested HIV-negative), 96% (newly tested HIV-positive), and 100% (established HIV infection). Those newly testing HIV-negative and HIV-positive were younger on average (mean 32.2 years and 37.5 years, respectively) than the community sample (mean 39.6 years), while those with established HIV infection were older (mean 42.6 years). Those newly testing HIV-negative were evenly divided between males and females, whereas about two-thirds of those newly testing HIV-positive and those with established HIV infection were female. The newly tested HIV-negative individuals were comparable in physical and mental health scores to the community sample with the exception of elevated PTSD symptom severity. Those newly testing HIV-positive and those with established HIV infection had worse overall physical and mental health scores and greater PTSD symptom severity compared to the community cohort, and those newly testing HIV-positive also had elevated depressive symptoms compared to the other cohorts.

### Traumatic and Stressful Events

Those with established HIV infection had the highest exposure to childhood (mean: 2.1 types of trauma) and lifetime (mean: 3.0) potentially traumatic experiences (Table 1). The exposure of those newly testing HIV-positive (childhood: 1.7; lifetime: 2.2) was comparable to the community cohort (childhood: 1.8; lifetime: 2.3), whereas those newly testing HIV-negative had lower exposure than the community cohort (childhood: 1.5; lifetime: 1.9). Recent stressful life events were comparable between the community cohort (mean: 4.2 events in past 6 months) and those with established HIV infection (4.1 events), while those newly testing HIV-negative (3.2 events) and HIV-positive (2.9 events) had fewer recent stressful life events.

The most common type of potentially traumatic experience in all cohorts was childhood physical neglect, reported by over half of the community cohort and those with established HIV infection and by one-third of those newly testing HIV-negative and HIV-positive (Table 2). Other common potentially traumatic experiences from childhood included parental use of alcohol or drugs in the home, reported by one-quarter to one-half of all cohorts, and death of a close relative, reported by one-quarter to one-third of all cohorts. Parental mental illness, suicide attempt, or successful suicide was reported by 4–7% of respondents, depending on the cohort.

**Table 2:** Prevalence of lifetime potentially traumatic experiences.

	n (%)			
Experience	Community	Newly tested HIV-	Newly diagnosed HIV+	Established HIV+
				
Childhood experiences				
Sexual abuse before puberty	18 (7.3)	16 (9.0)	29 (11.0)	13 (6.0)
Physical abuse before puberty	5 (2.0)	8 (4.5)	7 (2.7)	5 (2.3)
Parental alcoholism or use of drugs	89 (35.7)	49 (26.9)	75 (28.1)	133 (58.3)**
Parental mental illness, suicide, or suicide attempt	15 (6.0)	11 (6.0)	19 (7.1)	8 (3.5)
Parental imprisonment	12 (4.8)	5 (2.7)	9 (3.4)	12 (5.3)
Parental fighting and threats	44 (17.7)	27 (14.8)	44 (16.5)	69 (30.3)**
Emotional neglect	32 (13.1)	17 (9.6)	73 (27.7)**	27 (12.4)
Physical neglect	155 (62.8)	59 (33.3)**	95 (36.0)**	115 (53.0)*
Time in orphanage	3 (1.2)	3 (1.6)	1 (0.4)	2 (0.9)
Time in jail	2 (0.8)	5 (2.7)	11 (4.1)*	4 (1.8)
Life-threatening illness	22 (8.8)	10 (5.5)	9 (3.4)**	12 (5.3)
Death of close relative	58 (23.3)	58 (31.9)*	75 (28.1)	66 (28.9)
Lifetime experiences				
Sexual abuse since puberty	20 (8.1)	5 (2.8)*	19 (7.2)	16 (7.4)
Physical abuse or assault since puberty	24 (9.8)	13 (7.4)	16 (6.1)	14 (6.5)
Death of a close person due to other people^1^	40 (16.1)	8 (4.4)**	8 (3.0)**	20 (8.8)*
Death of a child	34 (13.7)	29 (15.9)	41 (15.4)	58 (25.4)**
Death of spouse or partner	16 (6.4)	16 (8.8)	48 (18.0)**	73 (32.0)**

1 Murder, reported witchcraft, or traffic accident.

* p<0.05, **p<0.01 comparing each cohort to the Community cohort using a Chi-square test.

In adulthood, 25% of those with established HIV infection and 14–15% of those in the other cohorts had lost a child. In addition, 32% of those with established HIV infection and 18% of those newly testing HIV-positive had lost a spouse or partner, compared to 6–9% of those in the community cohort or newly testing HIV-negative.

Sexual abuse before puberty was reported by 6–11% of respondents, depending on the cohort, and sexual abuse after puberty was reported by 3–8% of respondents. About one-third of those reporting sexual abuse before puberty also suffered sexual abuse after puberty. Severe physical abuse before puberty was reported by 2–5% of respondents, while physical abuse or assault after puberty was reported by 6–10% of respondents.

### Trauma History and Current Health

Greater exposure to potentially traumatic experiences was associated with worse self-reported mental health and health-related physical functioning measures (Table 3). Controlling for cohort fixed effects, in bivariate analyses each additional lifetime potentially traumatic experience was associated with an increase of 0.6 (95% CI: 0.2, 0.9) units on the PTSD symptomatology scale, an increase of 0.5 (0.2, 0.7) units on the PHQ-9 depressive symptoms scale, and a decreases of −0.7 (−1.1, −0.2) units on the SF-8 health-related physical functioning composite score. When attention was restricted to childhood traumatic experiences, similar results were observed. Recent stressful life events were also associated with worse mental health and health-related physical functioning. Each additional recent stressful event was associated with an increase of 1.0 (95% CI: 0.8, 1.2) unit on the PTSD symptomatology scale, an increase of 0.6 (0.4, 0.7) units on the PHQ-9 depressive symptoms scale, and a decrease of 0.5 (−0.7, −0.2) units on the SF-8 health-related physical functioning score.

**Table 3:** Bivariate association of lifetime trauma exposure with mental and physical health.

	PTSDSymptoms(range: 17–85)	DepressiveSymptoms(range: 0–27)	Health-relatedphysical functioning(range: 0–100)
Number of childhood traumatic experiences	0.56 (0.22, 0.91)**	0.46 (0.20, 0.73)**	−0.68 (−1.13, −0.24)**
Number of lifetime traumatic experiences	0.68 (0.38, 0.97)**	0.49 (0.26, 0.72)**	−0.64 (−1.03, −0.26)**
Number of stressful life events in past 6 months	0.97 (0.78, 1.16)**	0.58 (0.43, 0.72)**	−0.45 (−0.70, −0.19)**

* p<0.05,

** p<0.01.

For PTSD and depressive symptoms, a higher score indicates worse health. For the SF8 health-related physical functioning score, a lower score indicates worse functioning.

Results are presented as ordinary least squares regression coefficient (95% confidence interval) per 1-unit increase in the independent variable.

All models included fixed effects for cohorts. In all models, we tested for homogeneity of associations across cohorts and linearity of the association of the dependent and independent variables.

Models to test linearity generally supported linear relationships between the traumatic and stressful event measures and health status, indicating progressive worsening of physical or mental health with each additional potentially traumatic or stressful event. An exception was the relationship between recent stressful events and PTSD symptomatology, for which a quadratic model suggested the increase in PTSD symptoms accelerated as the number of recent stressful events increased. Models that used interaction terms to test for homogeneity across cohorts in the relationships between the traumatic and stressful event measures and health status supported homogeneous relationships across cohorts (P values for likelihood ratio tests comparing nested models with and without interaction terms >0.05).

Table 4 presents a series of multivariate regression models examining the change in the magnitude of the association of trauma history with PTSD symptomatology as two hypothesized mediators (recent stressful events and current depressive symptoms) are added to the model. In Model 1, without mediators, trauma history remained associated with increased PTSD symptomatology after adjusting for demographic and socio-economic characteristics (Table 4, Model 1). Women reported greater PTSD symptomatology than men, and greater household assets were associated with fewer PTSD symptoms. When the number of recent stressful events was added to the model (Table 4, Model 2), the coefficient for trauma was attenuated by 41% but remained statistically significant, suggesting that recent stressful events might moderate part – but not all – of the relationship between history of potentially traumatic experiences and current PTSD symptoms. When current depressive symptoms were added to the model (Table 4, Model 3), the coefficient for trauma was attenuated an additional 20% and the coefficient for recent stressful events was attenuated by 23%, suggesting that current depressive symptoms might moderate part – but not all – of the relationships between trauma, stressful events, and current PTSD symptoms.

**Table 4:** Multivariate model of predictors of PTSD symptomatology.

	Model 1	Model 2	Model 3
Number of lifetime traumatic experiences	0.633 (0.326, 0.940)**	0.375 (0.077, 0.673)*	0.252 (−0.034, 0.537)
Number of stressful life events in past 6 months	NA	0.932 (0.738, 1.126)**	0.718 (0.527, 0.909)**
Depressive symptoms	NA	NA	0.385 (0.304, 0.465)**
Age, per 10 years	−0.169 (−0.681, 0.343)	−0.051 (−0.540, 0.439)	−0.089 (−0.556, 0.378)
Female gender	1.345 (0.294, 2.395)*	1.508 (0.506, 2.510)**	1.532 (0.576, 2.489)**
Marital status			
Married	ref	ref	ref
Single	−0.272 (−1.658, 1.113)	0.527 (−0.806, 1.859)	0.304 (−0.968, 1.577)
Widowed	−0.045 (−1.642, 1.551)	0.567 (−0.960, 2.095)	0.274 (−1.186, 1.733)
Divorced	0.300 (−1.202, 1.802)	0.761 (−0.674, 2.196)	0.023 (−1.355, 1.402)
Educational attainment			
None	ref	ref	ref
Primary	−1.341 (−4.191, 1.508)	−1.981 (−4.702, 0.740)	−1.651 (−4.249, 0.947)
Secondary	−0.296 (−3.391, 2.799)	−1.324 (−4.284, 1.635)	−1.186 (−4.011, 1.639)
University	0.619 (−3.521, 4.760)	0.026 (−3.925, 3.977)	−0.966 (−4.743, 2.810)
Household assets	−0.487 (−0.861, −0.112)*	−0.424 (−0.781, −0.066)*	−0.264 (−0.607, 0.079)
Cohort			
Community	ref	ref	ref
Newly tested HIV-	3.452 (1.930, 4.973)**	4.084 (2.627, 5.540)**	3.890 (2.499, 5.281)**
Newly diagnosed HIV+	5.658 (4.292, 7.023)**	6.596 (5.279, 7.913)**	5.018 (3.718, 6.318)**
Established HIV+	1.239 (−0.195, 2.673)	1.074 (−0.294, 2.442)	0.982 (−0.324, 2.288)
Model fit statistics			
F statistic (d.f.); P value	9.11 (13, 882); <0.0001	15.63 (14, 881); <0.0001	21.82 (15, 880); <0.0001
R^2^	0.12	0.20	0.27

* p<0.05,

** p<0.01.

Results are presented as Coefficient (95% confidence interval), where the coefficient represents the predicted change in the PTSD scale score (possible range: 17–85) for each one-unit change in the predictor variable. The successive models show the attenuation of the association of trauma with the outcome as successive hypothesized mediators are added to the model. Model 1 shows the association of number of lifetime traumatic experiences with PTSD symptomatology, adjusted for sociodemographic characteristics and cohort fixed effects. Model 2 shows the same association after additionally adjusting for recent stressful life events, and Model 3 shows the same association after additionally adjusting for current depressive symptoms.

**Table 5:** Multivariate model of health-related physical functioning.

	Model 1	Model 2	Model 3	Model 4
Number of lifetime traumatic experiences	−0.607 (−1.006, −0.208)**	**−**0.485 (**−**0.888, **−**0.082)*	**−**0.223 (**−**0.569, 0.123)	**−**0.183 (**−**0.532, 0.166)
Number of stressful life eventsin past 6 months	NA	**−**0.443 (**−**0.707, **−**0.179)**	0.045 (**−**0.188, 0.277)	0.089 (**−**0.152, 0.329)
Depressive symptoms	NA	NA	**−**0.897 (**−**0.995, **−**0.799)**	**−**0.876 (**−**0.980, **−**0.772)**
PTSD symptoms	NA	NA	NA	**−**0.065 (**−**0.146, 0.016)
Age, per 10 years	**−**0.670 (**−**1.337, **−**0.004)*	**−**0.722 (**−**1.386, **−**0.058)*	**−**0.694 (**−**1.262, **−**0.126)*	**−**0.693 (**−**1.263, **−**0.122)*
Female gender	**−**1.469 (**−**2.839, **−**0.099)*	**−**1.547 (**−**2.910, **−**0.183)*	**−**1.606 (**−**2.773, **−**0.439)**	**−**1.509 (**−**2.684, **−**0.333)*
Marital status				
Married	ref	ref	ref	ref
Single	**−**0.704 (**−**2.509, 1.102)	**−**1.083 (**−**2.892, 0.727)	**−**0.614 (**−**2.164, 0.935)	**−**0.572 (**−**2.127, 0.982)
Widowed	0.784 (**−**1.299, 2.868)	0.498 (**−**1.581, 2.577)	1.210 (**−**0.571, 2.991)	1.180 (**−**0.603, 2.963)
Divorced	**−**0.741 (**−**2.702, 1.219)	**−**0.953 (**−**2.907, 1.000)	0.750 (**−**0.933, 2.432)	0.725 (**−**0.959, 2.410)
Educational attainment				
None	ref	ref	ref	ref
Primary	3.512 (**−**0.210, 7.234)	3.812 (0.106, 7.518)*	3.029 (**−**0.144, 6.202)	2.957 (**−**0.219, 6.133)
Secondary	1.922 (**−**2.121, 5.965)	2.413 (**−**1.619, 6.444)	2.046 (**−**1.405, 5.497)	1.995 (**−**1.457, 5.447)
University	0.821 (**−**4.589, 6.231)	1.105 (**−**4.278, 6.487)	3.389 (**−**1.225, 8.002)	3.352 (**−**1.262, 7.966)
Household assets	0.569 (0.081, 1.057)**	0.539 (0.053, 1.025)**	0.151 (**−**0.267, 0.569)**	0.141 (**−**0.278, 0.560)
Cohort				
Community	ref	ref	ref	ref
Newly tested HIV-	**−**0.289 (**−**2.273, 1.695)**	**−**0.581 (**−**2.562, 1.400)**	**−**0.265 (**−**1.960, 1.431)**	0.012 (**−**1.717, 1.740)
Newly diagnosed HIV+	−5.896 (−7.675, −4.118)**	−6.342 (−8.130, −4.553)**	−2.802 (−4.381, −1.224)**	−2.399 (−4.038, −0.760)**
Established HIV+	−4.133 (−6.003, −2.263)**	−4.058 (−5.918, −2.197)**	−3.873 (−5.466, −2.280)**	−3.769 (−5.366, −2.171)**
Model fit statistics				
F statistic (d.f.); P value	8.22 (13, 885); <0.0001	8.49 (14, 884); <0.0001	32.41 (15, 883); <0.0001	30.46 (16, 879); <0.0001
R^2^	0.11	0.12	0.36	0.36

* p<0.05,

** p<0.01.

Results are presented as Coefficient (95% confidence interval), where the coefficient represents the predicted change in the SF-8 physical composite score (possible range: 0–100) for each one-unit change in the predictor variable. The successive models show the attenuation of the association of trauma with the outcome as successive hypothesized mediators are added to the model. Model 1 shows the association of number of lifetime traumatic experiences with current health-related physical functioning, adjusted for sociodemographic characteristics and cohort fixed effects. Model 2 shows the same association after additionally adjusting for recent stressful life events, Model 3 shows the same association after additionally adjusting for current depressive symptoms, and Model 4 shows the same association after additionally adjusting for current PTSD symptoms.

In a similar set of nested models with the SF-8 health-related physical functioning score as the outcome, history of potentially traumatic experiences was associated with worse health-related physical functioning after adjusting for demographic and socio-economic characteristics (Table 5, Model 1). Women had worse health-related physical functioning than men, and greater household assets were associated with improved health-related physical functioning. When the number of recent stressful events was added to the model (Table 5, Model 2), the coefficient for trauma was attenuated by 20% but remained statistically significant, suggesting that recent stressful events might moderate part – but not all – of the relationship between history of potentially traumatic experiences and current health-related physical functioning. When current depressive symptoms were added to the model (Table 5, Model 3), the coefficient for trauma was attenuated an additional 43% and the coefficient for recent stressful events was attenuated by 90%, suggesting that current depressive symptoms might moderate most or all of the relationships between trauma, stressful events, and current health-related physical functioning. The further addition of PTSD symptomatology to the model did not substantively change other coefficients (Table 5, Model 4).

## Discussion

This study found that Tanzanian adults with established HIV infection had higher exposure to both childhood and lifetime potentially traumatic experiences than did the newly tested HIV positive, newly tested HIV negative, or community samples. The newly tested HIV positive and community cohorts were similar, and those newly tested HIV negative had the lowest overall lifetime exposure to potentially traumatic events. These findings are similar to Western studies demonstrating higher prevalence of exposure to traumatic events among PLWHA. [7], [10], [23], [63] However, this excess was not due to higher prevalence of childhood sexual and physical abuse: the reported prevalence of these experiences among the cohort with established HIV infection (6% and 2%, respectively) was lower than in many other studies and was not elevated relative to the geographically matched adult community cohort. The higher overall trauma exposure among those with established HIV infection relative to the community cohort in this study was due primarily to higher prevalence during childhood of parental alcohol use, drug use, fighting, or threats, and higher prevalence of loss of a child or spouse. Some of these events, such as childhood experiences, are likely to have preceded HIV infection; others, such as loss of a child or spouse, may partially reflect higher incidence of HIV infection among spouses or children. Exposure to potentially traumatic events in childhood is associated with high risk sexual and drug use behaviors that increase risk of acquiring HIV, so those who experienced more childhood trauma may have, through a history of increased risk behaviors, contracted HIV earlier in life, thereby placing them in the established HIV infection rather than newly diagnosed HIV cohort. [14], [22]–[28].

In bivariate analyses, we determined that greater exposure to potentially traumatic events was associated with poorer physical and mental health according to patient self-report measures. There was a linear relationship between number of lifetime potentially traumatic events and PTSD and depressive symptom severity, as well as health-related physical functioning scores. Multivariate models confirmed trauma history as associated with increased PTSD symptom severity. The association with trauma was attenuated, but not completely explained, by recent stressful events and presence of current depressive symptoms. Similarly, multivariate models of the association of lifetime traumatic event exposure with health-related physical functioning demonstrated that exposure to potentially traumatic experiences was associated with worse health-related physical functioning. In this case, the addition of recent stressful events and current depressive and PTSD symptoms to the model appeared to completely explain the association, which suggests that current mental health symptoms may moderate complex relationships between trauma, stress, and current health-related physical functioning. These findings are consistent with previous research which identified associations between trauma history and health-related physical functioning in PLWHA that was partially or fully mediated by current mental health indicators. [31].

To date, the majority of sub-Saharan African studies looking at prevalence of exposure to traumatic events have occurred in populations exposed to large-scale conflict and displacement. [64], [65] Data about the prevalence of traumatic life events in the general population in sub-Saharan Africa or other low income nations are much more limited. One exception is a longitudinal study of South African women which showed that those with a history of intimate partner violence had a greater risk of HIV acquisition than those without. [64] In a second study comparing trauma history and PTSD prevalence between PLWHA in the US Deep South and Tanzania, one fourth of Tanzanian respondents compared to 36% of US respondents reported a history of sexual assault; 7% of U.S. respondents had a probable diagnosis of PTSD compared to 22% of Tanzanian respondents. [65] Given the clear effects of trauma on mental health, health-related physical functioning, HIV risk behaviors, and HIV disease progression in Western, conflict-exposed, and refugee populations, it is essential to better characterize the prevalence of exposure to potentially traumatic events in PLWHA and their influence on health indicators and other important outcomes, especially as access to HIV treatment programs in sub-Saharan Africa is rapidly expanding.

In the U.S., history of exposure to traumatic events is associated with poor treatment adherence, high risk sexual and drug use behaviors that increase disease transmission, lower medication adherence, and increased rates of progression to AIDS and mortality. [14], [22]–[28], [31]–[34] Further, in U.S. populations, exposure to potentially traumatic events appears to have a cumulative effect, in which people with higher trauma exposure experience poorer outcomes, including lower ART adherence, [66] higher virologic failure, [66] and increased AIDS-related and all-cause mortality rates. [33] These results are consistent with the findings from the present study that current mental health and health-related physical functioning appeared to deteriorate in a linear fashion with increasing trauma exposure. Interestingly, in one study patients with a diagnosis of PTSD related to their HIV diagnosis actually had lower rates of suboptimal adherence to ART. [67] Patients with PTSD related to potentially traumatic events other than HIV diagnosis were not included, and it is likely that the anxiety and distress related to PTSD, since centered around fear of HIV and disease progression, may contribute to medication adherence. However, in PLWHA who have a diagnosis of PTSD as a result of any type of trauma, ART adherence is lower; findings which are in keeping with the body of literature on PTSD and ART adherence in PLWHA. [68]–[70].

In interpreting the results of this study, it should be noted that exposure to traumatic events, depressive and PTSD symptoms, and health-related physical functioning were measured by self-report. Reporting of prior traumatic events may have been subject to recall bias because most traumatic events occurred years prior to the study interviews, or under-reporting due to reluctance to mention difficult experiences. It is possible that recall of past events was differential in ways that would bias some of the associations examined in this paper, for example if those with worse current mental health were more likely to recall difficult past experiences. Thus this study may have underestimated the prevalence of potentially traumatic events. In addition, the cross-sectional design of the analyses presented here limits the ability to make causal inferences about the relationships between exposure to traumatic events and changes in mental health and health-related physical functioning. We estimated the prevalence of trauma in HIV patients in established medical care, but did not include HIV-infected individuals who were not in care, which may have led to an underestimation of the mental health burden among HIV-infected individuals overall. Finally, a number of comparisons are reported, suggesting caution in interpreting indications of statistical significance. One notable strength of the study is the inclusion of a geographically matched community cohort; most previous studies have compared trauma rates to general population rates rather than to rates in the HIV-positive participants’ communities of origin.

Our findings suggest that the associations between potentially traumatic events and current mental health and health-related physical functioning in PLWHA which have been observed in high income countries may apply in sub-Saharan African populations as well. These results underline the importance of identifying and responding to trauma histories within HIV medical care. As access to HIV medical care and antiretroviral therapy continues to expand rapidly in sub-Saharan Africa, further research is needed in the region to confirm whether trauma predicts other HIV-related behaviors and outcomes of epidemiologic and clinical importance, such as sexual risk behaviors, engagement in medical care, medication adherence, and mortality, as it has in high income countries. At the same time, consideration must be given now to designing HIV clinical programs capable of addressing psychosocial characteristics that may enhance or impede engagement in care and clinical response.

## References

[1] Whetten K, Reif S, Whetten R, Murphy-McMillan LK (2008). Trauma, mental health, distrust, and stigma among HIV-positive persons: implications for effective care.. Psychosom Med.

[2] Kilpatrick DG, Saunders BE (2000). https://www.ncjrs.gov/pdffiles1/nij/grants/181028.pdf.

[3] Tjaden P, Thoennes N (2000). http://www.nij.gov/pubs-sum/181867.htm.

[4] Whetten K, Leserman J, Lowe K, Stangl D, Thielman N (2006). Prevalence of Childhood Sexual Abuse and Physical Trauma in an HIV-Positive Sample From the Deep South.. Am J Public Health.

[5] Pence BW, Reif S, Whetten K, Leserman J, Stangl D (2007). Minorities, the poor, and survivors of abuse: HIV-infected patients in the US deep South.. South Med J.

[6] Wyatt GE, Myers HF, Loeb TB (2004). Women, Trauma, and HIV: an overview.. AIDS Behav.

[7] Kalichman SC, Sikkema KJ, DiFonzo K, Luke W, Austin J (2002). Emotional adjustment in survivors of sexual assault living with HIV-AIDS.. J Trauma Stress.

[8] Epstein JN, Saunders BE, Kilpatrick DG (1997). Predicting PTSD in women with a history of childhood rape.. J Trauma Stress.

[9] Rodriguez N, Ryan SW, Vande Kemp H, Foy DW (1997). Posttraumatic stress disorder in adult female survivors of childhood sexual abuse: a comparison study.. J Consult Clin Psychol.

[10] Roth S, Newman E, Pelcovitz D, van der Kolk B, Mandel FS (1997). Complex PTSD in victims exposed to sexual and physical abuse: results from the DSM-IV Field Trial for Posttraumatic Stress Disorder.. J Trauma Stress.

[11] Zlotnick C (1997). Posttraumatic stress disorder (PTSD), PTSD comorbidity, and childhood abuse among incarcerated women.. J Nerv Ment Dis.

[12] Epstein JN, Saunders BE, Kilpatrick DG, Resnick HS (1998). PTSD as a mediator between childhood rape and alcohol use in adult women.. Child Abuse Negl.

[13] Widom CS (1999). Posttraumatic stress disorder in abused and neglected children grown up.. Am J Psychiatry.

[14] McCauley J, Kern DE, Kolodner K, Dill L, Schroeder AF (1997). Clinical characteristics of women with a history of childhood abuse: unhealed wounds.. Jama.

[15] de Paul J, Domenech L (2000). Childhood history of abuse and child abuse potential in adolescent mothers: a longitudinal study.. Child Abuse Negl.

[16] Styron T, Janoff-Bulman R (1997). Childhood attachment and abuse: long-term effects on adult attachment, depression, and conflict resolution.. Child Abuse Negl.

[17] Allers CT, Benjack KJ (1991). Connections Between Childhood Abuse and HIV Infection.. J Couns Dev.

[18] Wenninger K, Ehlers A (1998). Dysfunctional cognitions and adult psychological functioning in child sexual abuse survivors.. J Trauma Stress.

[19] Kunitz SJ, Levy JE, McCloskey J, Gabriel KR (1998). Alcohol dependence and domestic violence as sequelae of abuse and conduct disorder in childhood.. Child Abuse Negl.

[20] Fleming J, Mullen PE, Sibthorpe B, Bammer G (1999). The long-term impact of childhood sexual abuse in Australian women.. Child Abuse Negl.

[21] Merrill LL, Newell CE, Thomsen CJ, Gold SR, Milner JS (1999). Childhood abuse and sexual revictimization in a female Navy recruit sample.. J Trauma Stress.

[22] Hamburger ME, Moore J, Koenig LJ, Vlahov D, Schoenbaum EE (2004). Persistence of inconsistent condom use: relation to abuse history and HIV serostatus.. AIDS Behav.

[23] Kelly JA, Murphy DA, Bahr GR, Koob JJ, Morgan MG (1993). Factors associated with severity of depression and high-risk sexual behavior among persons diagnosed with human immunodeficiency virus (HIV) infection.. Health Psychol.

[24] Hutton HE, Treisman GJ, Hunt WR, Fishman M, Kendig N (2001). HIV risk behaviors and their relationship to posttraumatic stress disorder among women prisoners.. Psychiatr Serv.

[25] Springs FE, Friedrich WN (1992). Health risk behaviors and medical sequelae of childhood sexual abuse.. Mayo Clin Proc.

[26] Bensley LS, Van Eenwyk J, Simmons KW (2000). Self-reported childhood sexual and physical abuse and adult HIV-risk behaviors and heavy drinking.. Am J Prev Med.

[27] Parillo KM, Freeman RC, Collier K, Young P (2001). Association between early sexual abuse and adult HIV-risky sexual behaviors among community-recruited women.. Child Abuse Negl.

[28] Paolucci EO, Genuis ML, Violato C (2001). A meta-analysis of the published research on the effects of child sexual abuse.. J Psychol.

[29] Israelski DM, Prentiss DE, Lubega S, Balmas G, Garcia P (2007). Psychiatric co-morbidity in vulnerable populations receiving primary care for HIV/AIDS.. AIDS Care.

[30] Myer L, Smit J, Roux LL, Parker S, Stein DJ (2008). Common mental disorders among HIV-infected individuals in South Africa: prevalence, predictors, and validation of brief psychiatric rating scales.. AIDS Patient Care STDS.

[31] Leserman J, Whetten K, Lowe K, Stangl D, Swartz MS (2005). How trauma, recent stressful events, and PTSD affect functional health status and health utilization in HIV-infected patients in the south.. Psychosom Med.

[32] Mugavero MJ, Pence BW, Whetten K, Leserman J, Swartz M (2007). Predictors of AIDS-related morbidity and mortality in a southern U.S. Cohort.. AIDS Patient Care STDS.

[33] Leserman J, Pence BW, Whetten K, Mugavero MJ, Thielman NM (2007). Relation of lifetime trauma and depressive symptoms to mortality in HIV.. Am J Psychiatry.

[34] Mugavero M, Ostermann J, Whetten K, Leserman J, Swartz M (2006). Barriers to antiretroviral adherence: the importance of depression, abuse, and other traumatic events.. AIDS Patient Care STDS.

[35] Gill CJ, Griffith JL, Jacobson D, Skinner S, Gorbach SL (2002). Relationship of HIV viral loads, CD4 counts, and HAART use to health-related quality of life.. J Acquir Immune Defic Syndr.

[36] Campsmith ML, Nakashima AK, Davidson AJ (2003). Self-reported health-related quality of life in persons with HIV infection: results from a multi-site interview project.. Health Qual Life Outcomes.

[37] Dobalian A, Tsao JC, Duncan RP (2004). Pain and the use of outpatient services among persons with HIV: results from a nationally representative survey.. Med Care.

[38] Jacobson DL, Wu AW, Feinberg J (2003). Health-related quality of life predicts survival, cytomegalovirus disease, and study retention in clinical trial participants with advanced HIV disease.. J Clin Epidemiol.

[39] UNAIDS, WHO (2009). http://www.unaids.org/en/dataanalysis/epidemiology/2009aidsepidemicupdate/.

[40] Olley BO, Seedat S, Stein DJ (2006). Persistence of psychiatric disorders in a cohort of HIV/AIDS patients in South Africa: a 6-month follow-up study.. J Psychosom Res.

[41] Martin L, Fincham D, Kagee A (2009). Screening for HIV-related PTSD: sensitivity and specificity of the 17-item Posttraumatic Stress Diagnostic Scale (PDS) in identifying HIV-related PTSD among a South African sample.. Afr J Psychiatry (Johannesbg).

[42] Martin L, Kagee A (2011). Lifetime and HIV-related PTSD among persons recently diagnosed with HIV.. AIDS Behav.

[43] Ware JE, Kosinski M, Bayliss MS, McHorney CA, Rogers WH (1995). Comparison of methods for the scoring and statistical analysis of SF-36 health profile and summary measures: summary of results from the Medical Outcomes Study.. Med Care.

[44] Ware JE, Sherbourne CD (1992). The MOS 36-item short-form health survey (SF-36). I. Conceptual framework and item selection.. Med Care.

[45] Ware JE, Kosinski M, Dewey JE (2001). How to score and interpret single-item health status measures: a manual for users of the SF-8™ Health Survey..

[46] Adewuya AO, Ola BA, Afolabi OO (2006). Validity of the patient health questionnaire (PHQ-9) as a screening tool for depression amongst Nigerian university students.. J Affect Disord.

[47] Monahan PO, Shacham E, Reece M, Kroenke K, Ong’or WO (2009). Validity/Reliability of PHQ-9 and PHQ-2 Depression Scales Among Adults Living with HIV/AIDS in Western Kenya.. J Gen Intern Med.

[48] Kroenke K, Spitzer RL, Williams JB (2001). The PHQ-9: validity of a brief depression severity measure.. J Gen Intern Med.

[49] Lowe B, Unutzer J, Callahan CM, Perkins AJ, Kroenke K (2004). Monitoring depression treatment outcomes with the patient health questionnaire-9.. Med Care.

[50] Weathers F, Ford J, Stamm B (1996). Psychometric properties of the PTSD Checklist.. Measurement of stress, trauma, and adaptation.

[51] Blanchard EB, Jones-Alexander J, Buckley TC, Forneris CA (1996). Psychometric properties of the PTSD Checklist (PCL).. Behav Res Ther.

[52] American Psychiatric Association (2000). Diagnostic and Statistical Manual of Mental Disorders..

[53] Bernstein D, Fink, L (1998). Childhood Trauma Questionnaire Manual..

[54] Kilpatrick D, Resnick H, Davidson JRT, Foa EB (1993). A description of the posttraumatic stress disorder field trial.. Posttraumatic stress disorder: DSM-IV and beyond.

[55] Koss MP, Gidycz CA (1985). Sexual experiences survey: reliability and validity.. J Consult Clin Psychol.

[56] Leserman J, Li Z, Drossman DA, Toomey TC, Nachman G (1997). Impact of sexual and physical abuse dimensions on health status: development of an abuse severity measure.. Psychosom Med.

[57] Dube SR, Felitti VJ, Dong M, Chapman DP, Giles WH (2003). Childhood abuse, neglect, and household dysfunction and the risk of illicit drug use: the adverse childhood experiences study.. Pediatrics.

[58] Felitti VJ, Anda RF, Nordenberg D, Williamson DF, Spitz AM (1998). Relationship of childhood abuse and household dysfunction to many of the leading causes of death in adults. The Adverse Childhood Experiences (ACE) Study.. Am J Prev Med.

[59] Liebschutz JM, Feinman G, Sullivan L, Stein M, Samet J (2000). Physical and sexual abuse in women infected with the human immunodeficiency virus: increased illness and health care utilization.. Arch Intern Med.

[60] Sarason IG, Johnson JH, Siegel JM (1978). Assessing the impact of life changes: development of the Life Experiences Survey.. J Consult Clin Psychol.

[61] Leserman J, Ironson G, O’Cleirigh C, Fordiani JM, Balbin E (2008). Stressful life events and adherence in HIV.. AIDS Patient Care STDS.

[62] Leserman J, Petitto JM, Gu H, Gaynes BN, Barroso J (2002). Progression to AIDS, a clinical AIDS condition and mortality: psychosocial and physiological predictors.. Psychol Med.

[63] Basile KC, Chen J, Black MC, Saltzman LE (2007). Prevalence and characteristics of sexual violence victimization among U.S. adults, 2001–2003.. Violence Vict.

[64] Jewkes RK, Dunkle K, Nduna M, Shai N (2010). Intimate partner violence, relationship power inequity, and incidence of HIV infection in young women in South Africa: a cohort study.. Lancet.

[65] Whetten K, Whetten RA, Ostermann J, Itemba D (2008). Trauma, anxiety and reported health among HIV-positive persons in Tanzania and the US Deep South.. AIDS Care.

[66] Mugavero MJ, Raper JL, Reif S, Whetten K, Leserman J (2009). Overload: impact of incident stressful events on antiretroviral medication adherence and virologic failure in a longitudinal, multisite human immunodeficiency virus cohort study.. Psychosom Med.

[67] Nilsson Schonnesson L, Williams ML, Ross MW, Bratt G, Keel B (2007). Factors associated with suboptimal antiretroviral therapy adherence to dose, schedule, and dietary instructions.. AIDS Behav.

[68] Boarts JM, Sledjeski EM, Bogart LM, Delahanty DL (2006). The differential impact of PTSD and depression on HIV disease markers and adherence to HAART in people living with HIV.. AIDS Behav.

[69] Delahanty DL, Bogart LM, Figler JL (2004). Posttraumatic stress disorder symptoms, salivary cortisol, medication adherence, and CD4 levels in HIV-positive individuals.. AIDS Care.

[70] Cohen MA, Alfonso CA, Hoffman RG, Milau V, Carrera G (2001). The impact of PTSD on treatment adherence in persons with HIV infection.. Gen Hosp Psychiatry.

